# Sinusoidal endothelial cells control liver inflammation and fibrosis

**DOI:** 10.1172/JCI206430

**Published:** 2026-06-01

**Authors:** Yingfen Chen, Yong He

**Affiliations:** 1 State Key Laboratory of Drug Research, Shanghai Institute of Materia Medica (SIMM), Chinese Academy of Sciences, Shanghai, China.; 2 University of Chinese Academy of Sciences, Beijing, China.

## Abstract

Liver fibrosis is a common pathological outcome of chronic liver disease and is driven by inflammatory responses. However, the early signals that initiate the inflammatory cascade remain poorly understood. Emerging evidence suggests that liver sinusoidal endothelial cells (LSECs) are not merely passive bystanders, but active regulators during liver fibrosis. In this issue of the *JCI*, Gan et al. demonstrated in multiple preclinical models that BRD4/PML-mediated super-enhancer activation in LSECs drives proinflammatory angiocrine signaling, thereby initiating liver fibrosis. Thus, targeting this endothelial axis may offer a promising therapeutic strategy for the treatment of liver fibrosis.

## Introduction

The global burden of chronic liver disease is rising, with cirrhosis being currently ranked as the 11^th^ and 14^th^ leading cause of death and morbidity worldwide, respectively (1). Globally, liver diseases cause approximately two million deaths annually and account for 4% of all deaths, largely owing to complications of cirrhosis and hepatocellular carcinoma (2). Liver fibrosis is a common wound-healing response to chronic injury and may lead to cirrhosis and liver failure. Although our understanding of the mechanisms underlying liver fibrosis has advanced considerably, antifibrotic therapies in clinical trials have so far yielded disappointing results. This underscores the urgent need to further dissect fibrogenesis by identifying its central drivers and initiating events.

Liver fibrogenesis is a dynamic and potentially reversible process that involves interactions among multiple cell types, including hepatic stellate cells (HSCs), hepatocytes, liver sinusoidal endothelial cells (LSECs), and inflammatory cells. It is characterized by chronic hepatocellular injury, endothelial barrier damage, inflammation, and excessive extracellular matrix (ECM) deposition (3). Among these processes, inflammation plays a central role in liver fibrogenesis, as danger signals released from dying cells, extrahepatic signals, and chemokine networks collectively drive immune cell infiltration into the injured liver (4). Nevertheless, the dominant cellular players that initiate and sustain inflammation during liver fibrosis remain incompletely understood.

## Pathological roles of LSECs in liver fibrogenesis

LSECs line the hepatic sinusoids and are characterized by the absence of an organized basement membrane and the presence of open fenestrae, which facilitate the efficient exchange of circulating substances between the bloodstream and hepatocytes and are essential for maintaining liver homeostasis (5). Importantly, LSECs are a heterogeneous cell population with distinct transcriptomic profiles across different spatial zones of the liver lobule. Given the central role of LSECs in sinusoidal homeostasis, LSEC dysfunction is increasingly recognized as an early unifying mechanism across different liver pathologies (6). Upon injury, LSECs gradually lose their specialized phenotype in a process termed sinusoidal capillarization that is marked by the loss of their characteristic fenestrae and the development of a basement membrane. Notably, LSEC capillarization can occur even before fibrosis develops and is associated with the acquisition of a profibrotic phenotype and the secretion of a wide spectrum of profibrotic factors (7). Capillarized LSECs can also undergo partial endothelial-to-mesenchymal transition during liver fibrosis, acquiring mesenchymal features and potentially promoting fibrosis through the production of ECM components, including collagen and other fibrosis-related proteins (8).

Accordingly, increasing efforts have focused on identifying endothelial regulators that govern LSEC dysfunction and liver fibrosis. Among these regulators, the transcription factors GATA binding protein 4 (GATA4) (9), zinc-finger E-box-binding homeobox 2 (ZEB2) (10), and runt-related transcription factor 3 (RUNX3) (11) have emerged as crucial regulators of LSEC homeostasis and antifibrotic function. Endothelial GATA4 suppresses profibrotic angiocrine signaling and protects against perisinusoidal liver fibrosis (9), whereas ZEB2 preserves hepatic angioarchitecture, and its loss renders LSECs more prone to capillarization and disrupts LSEC-HSC communication (10). Moreover, endothelial *Runx3* deficiency causes gradual spontaneous liver fibrosis secondary to LSEC dysfunction and exacerbates acute liver injury, partially through paracrine activation of HSCs (11). Apart from these transcription factors, other endothelial regulators also contribute to LSEC-HSC communication during fibrosis. Protein *O*-fucosyltransferase 1 (POFUT1), an essential regulator of NOTCH signaling, prevents liver fibrogenesis during chronic injury by repressing fibrinogen synthesis in LSECs, while its loss enhances NOTCH/HES1/STAT3 signaling and HSC activation (12). Ras-associated protein 1A (RAP1A), a small GTPase of the RAS superfamily, has also been identified as a protective endothelial regulator that attenuates sinusoidal capillarization and liver fibrosis by suppressing NOTCH activation (13).

**Figure 1:**
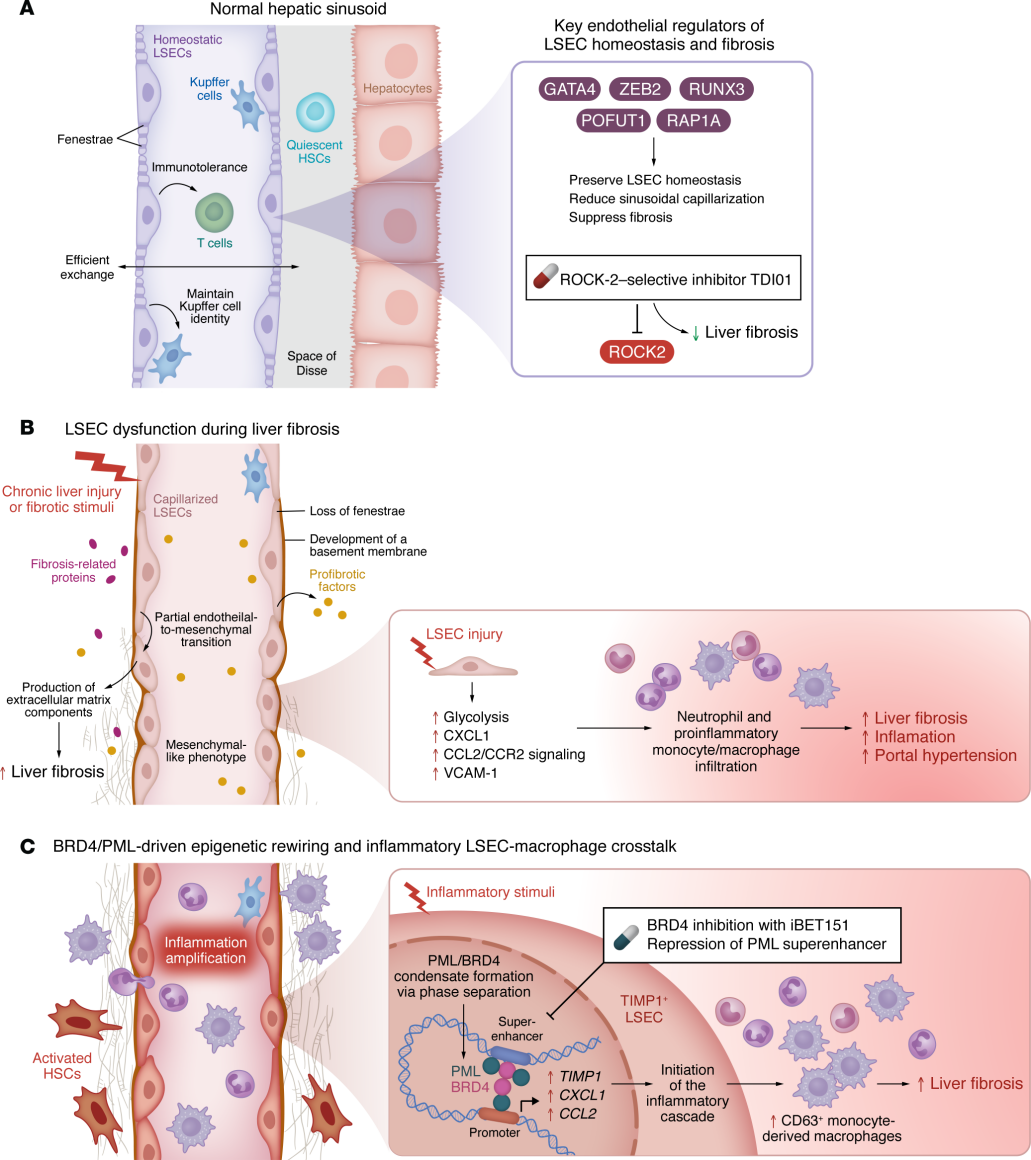
Roles of LSECs in liver fibrosis. (**A**) LSECs line the hepatic sinusoids and maintain liver homeostasis by facilitating substance exchange, preserving Kupffer cell identity, and supporting immune tolerance. Endothelial regulators, including GATA4, ZEB2, RUNX3, POFUT1, and RAP1A, help preserve LSEC homeostasis and restrain liver fibrosis, whereas ROCK2-selective inhibition with TDI01 represents a potential antifibrotic strategy. (**B**) Upon liver injury, loss of fenestrae and development of a basement membrane drive sinusoidal capillarization, and capillarized LSECs can further undergo partial endothelial-to-mesenchymal transition, resulting in enhanced profibrotic angiocrine signaling and excessive extracellular matrix deposition. Injured LSECs can also adopt a proinflammatory phenotype, with increased expression of CXCL1, CCL2, and VCAM-1, thereby contributing to inflammatory cell recruitment and fibrogenesis. (**C**) Notably, as Gan et al. (18) reported in this issue of the JCI, BRD4/PML-driven epigenetic rewiring in TIMP1^+^ LSECs promotes super-enhancer–dependent inflammatory gene activation, recruits CD63^+^ monocyte-derived macrophages, and amplifies inflammation, finally aggravating liver fibrosis.

In addition to maintaining liver homeostasis and mediating angiocrine communication with HSCs, LSECs are increasingly recognized as important immunoregulatory cells that orchestrate hepatic inflammation, thus influencing fibrogenesis. Under homeostatic conditions, fenestrated LSECs maintain Kupffer cell (KC) identity and contribute to the immunotolerance of CD8^+^ and CD4^+^ T cells (14). However, upon injury, LSECs can be reprogrammed toward a proinflammatory phenotype. For example, mechanotransduction-induced glycolysis epigenetically regulates a C-X-C motif chemokine ligand 1–dominant (CXCL1-dominant) angiocrine signaling program in LSECs (15). Inhibition of glycolysis reduces CXCL1 production and subsequently attenuates CXCL1-driven neutrophil infiltration, early fibrosis, and portal hypertension (15). Furthermore, C-C motif chemokine ligand 2 (CCL2), a key chemokine for monocyte recruitment, is upregulated in LSECs and promotes the recruitment of CCR2^+^ monocyte-derived macrophages (MoMFs) into the liver, contributing to liver fibrosis and portal hypertension (16). Likewise, in murine models of metabolic dysfunction–associated steatohepatitis (MASH), vascular cell adhesion molecule 1 (VCAM-1), an adhesion molecule upregulated in LSECs, promotes the infiltration of proinflammatory monocytes into the liver, thereby exacerbating MASH-associated liver inflammation and fibrosis (17). Taken together, these studies support an important role of LSECs in promoting fibrosis by orchestrating immune cell recruitment during liver injury (Figure 1).

## Endothelial BRD4/PML axis drives fibrogenesis by recruiting macrophages

While accumulating evidence supports an important role for LSECs in orchestrating immune cell recruitment during liver injury, the upstream endothelial programs that translate injury-related cues into proinflammatory signals and initiate the inflammatory cascade during fibrogenesis remain incompletely understood. In this issue of the *JCI*, Gan et al. (18) identified a proinflammatory angiocrine program in murine fibrotic LSECs that is enriched for genes involved in chemotaxis, leukocyte migration and adhesion, and mononuclear cell proliferation. These conclusions were further supported by evaluating liver macrophage and neutrophil infiltration via performing intravital imaging for F4/80 (a macrophage marker) and Ly6G (a neutrophil marker) in fibrotic and nonfibrotic mouse livers. The authors found that endothelial CCL2 was markedly induced by tumor necrosis factor α (TNF-α), whereas inhibitors targeting the epigenetic reader domain most effectively suppressed *Ccl2* gene expression, with bromodomain-containing protein 4 (BRD4) inhibitors showing the greatest potency. BRD4 was abundant in endothelial cells and correlated with liver fibrosis development in human livers. Integrated multi-omic analyses identified promyelocytic leukemia (PML), a multifunctional protein involved in DNA repair, apoptosis, and gene expression (19), as a BRD4-dependent target driven by super enhancers (SEs). Interestingly, PML, in turn, directly bound BRD4 in the nucleus and promoted BRD4 accumulation via phase separation, thus amplifying proinflammatory gene expression. Importantly, using LSEC-specific *Brd4*- and *Pml*-deficient mice, the authors further demonstrated that disruption of the BRD4/PML axis alleviated liver inflammation and fibrosis. Further scRNA-seq analysis of healthy and CCl_4_-induced fibrotic livers with and without LSEC-specific *Brd4* depletion, revealed that TIMP1^+^ LSECs recruited CD63^+^ MoMFs during liver fibrosis progression. To evaluate the therapeutic potential, the authors showed that both pharmacological BRD4 inhibition with iBET151 and epigenetic repression of PML mitigated liver inflammation and fibrosis (Figure 1).

The study by Gan et al. (18) convincingly describes the critical role of epigenetically rewired LSECs in promoting inflammation during liver fibrosis, suggesting that targeting the BRD4/PML-TIMP1 axis may represent a promising therapeutic strategy. Nevertheless, some issues warrant further investigation. Since both BRD4 (20) and PML (19) have broad biological functions, the safety and translational feasibility of targeting this pathway remain to be determined in humans. Although the authors provided supportive evidence in human cirrhotic liver sections and performed in vitro experiments in hLSECs, further validation in human cohorts and translational studies is needed. Additionally, because the study of Gan et al. (18) mainly relied on CCl_4_- and DDC-induced mouse models, whether this phase separation–dependent BRD4/PML axis also operates in liver fibrosis in alcohol-associated liver disease (ALD), MASH, metabolic dysfunction and alcohol-associated liver disease (MetALD), and viral hepatitis remains to be established.

## Targeting LSECs to treat liver fibrosis

Although Resmetirom (21) and Semaglutide (22) have shown histologic benefit in selected patients with MASH-associated fibrosis, there are still no broadly approved antifibrotic therapies for liver fibrosis across different etiologies. Pharmacotherapies for liver fibrosis may also benefit from targeting LSECs. Notably, a recent study identified endothelial and perivascular Rho-associated coiled-coil containing kinase 2 (ROCK2) as a vascular druggable target in liver fibrosis. In line with this, the ROCK2-selective inhibitor TDI01 showed a trend toward reducing liver fibrosis in five of six patients in an extended clinical study (*ChiCTR2400082056*) (23) (Figure 1). In three preclinical models of liver fibrosis, targeted spherical nucleic acid nanoparticles restored LSEC fenestrations, reversed capillarization, and significantly reduced fibrosis, while showing a favorable biosafety profile (24), supporting the feasibility of LSEC-targeted antifibrotic therapy. Interestingly, spatial transcriptomic analysis of healthy and fibrotic human livers revealed remodeling of macrophage populations during fibrotic injury (25). Gan et al. (18) further demonstrated that proinflammatory LSECs actively recruit CD63^+^ MoMFs during liver fibrosis progression. Therefore, specifically targeting key mediators of LSEC-macrophage crosstalk, such as the BRD4/PML-TIMP1 axis identified by Gan et al. (18), may provide another attractive strategy to tackle liver fibrosis. Further elucidation of the molecular mechanisms underlying LSEC-macrophage crosstalk may facilitate the development of antifibrotic therapies.

## Conclusion

Collectively, the data presented by Gan et al. (18) reveal that epigenetically rewired LSECs initiate profibrotic inflammation in liver fibrosis. The authors document a pathogenic role of the BRD4/PML-TIMP1 axis in driving liver fibrogenesis and also demonstrate that targeting this key axis ameliorates liver inflammation and fibrosis in preclinical models. Nevertheless, the clinical relevance of this signaling pathway remains to be established, particularly given the broad biological functions of BRD4 and PML.

## Conflict of interest

The authors have declared that no conflict of interest exists.

## Funding support

- The National Key Research and Development Program of China (2023YFA1800804 to YH).
- The Noncommunicable Chronic Diseases-National Science and Technology Major Project (2024ZD0530604 to YH).
- Strategic Priority Research Program of the Chinese Academy of Sciences (XDB0830402 to YH).

## Version 1 06/01/2026

Electronic publication
